# Tracking Vigilance While Driving: Pilot Study of Heart Rate Variability Classification Under a Controlled Sleep-Deprivation Protocol

**DOI:** 10.3390/s26196104

**Published:** 2026-09-26

**Authors:** James Elber Duverger, Eli Moser, Philippe Boudreau, Marie Claude Ouimet, Diane B. Boivin, Alireza Saidi

**Affiliations:** 1 Institut de Recherche Robert-Sauvé en Santé et en Sécurité du Travail, Montréal, QC H3A 3C2, Canada; james.elber-duverger@irsst.qc.ca; 2 Centre for Study and Treatment of Circadian Rhythms, Douglas Mental Health University Institute, Department of Psychiatry, McGill University, Montréal, QC H4H 1R3, Canada; eli.moser.comtl@ssss.gouv.qc.ca (E.M.); philippe.boudreau@douglas.mcgill.ca (P.B.); diane.boivin@douglas.mcgill.ca (D.B.B.); 3 Faculty of Medicine and Health Sciences, University of Sherbrooke, Longueuil, QC J4K 0A8, Canada; marie.claude.ouimet@usherbrooke.ca

**Keywords:** vigilance tracking, sleep deprivation, driving simulation, heart rate variability, HRV classification, constant routine protocol, machine learning, participant-independent validation, LOSO-CV

## Abstract

Decreased vigilance associated with drowsiness is a major cause of motor vehicle crashes, but its physiological characterization and detection remain difficult due to behavioral and environmental confounding factors. This study presents an original experimental framework combining a highly controlled sleep-deprivation protocol, repeated driving simulations, and a machine-learning-based heart rate variability (HRV) analysis to study the physiological and behavioral changes in vigilance during a prolonged period of wakefulness while driving. Twelve healthy young adults participated in a 30-h sleep-deprivation constant routine protocol that included driving simulations every two hours, with data collection: electrocardiography (ECG), subjective ratings of drowsiness, psychomotor vigilance test, and driving performance. The collected data was separated into two clearly identifiable vigilance states, a “Rested” and a “Tired” state, respectively, for the first and last driving sessions, of the protocol. Heart rate variability (HRV) features were extracted from ECG recordings, statistically analyzed, and submitted into supervised machine learning classifiers using participant-independent validation to assess their ability to distinguish between two experimentally defined states of vigilance. The accuracy of state separation was verified based on significant alterations observed in drowsiness ratings and psychomotor performance in the Tired compared to the Rested state. The variability in driving behavior, an indicator of impaired driving ability, increased significantly from the Rested compared to the Tired state. Several HRV features revealed significant differences between Rested and Tired states, with effect sizes ranging from moderate to large, observed for several metrics. Participant-independent machine learning analysis, namely Leave-One-Subject-Out Cross-Validation (LOSO-CV), showed that HRV metrics enable reliable discrimination between vigilance states, with all classifiers evaluated achieving average accuracy greater than 85%. This pilot study demonstrated that the proposed experimental framework, by reducing the major behavioral and environmental confounding factors, can generate two distinct states of vigilance at the physiological and behavioral levels, enabling reliable separation of experimental data, independent of any additional ground truths. The states separated by the framework enable the detection of decreased driving performance and efficiently label HRV data for vigilance classification. Nevertheless, the findings should be interpreted within the context of a pilot study conducted with a small cohort of healthy young adults.

## 1. Introduction

Reduced vigilance associated with drowsiness and fatigue at the wheel is a major contributor to motor vehicle crashes worldwide. Accurately quantifying the burden of accidents caused by drowsy driving remains a challenge, as driver fatigue is often underreported in accident databases. Published estimates suggest that fatigue or drowsiness may contribute to a significant proportion of serious and fatal traffic accidents, although the reported values vary across countries and study methodologies. In the United States, analyses conducted by the AAA Foundation for Traffic Safety indicate that drowsiness may be a factor in approximately 16 to 21% of fatal accidents. Similar concerns regarding drowsiness-related crashes have been reported in Canada, Europe, and Asia, where driver drowsiness is recognized as a major road safety issue requiring targeted prevention strategies [1,2,3,4]. Indeed, fatigue has consistently been linked to impaired cognitive performance, reduced psychomotor vigilance, longer reaction times, and an increased likelihood of missed responses [5,6,7]. It has also been shown that acute sleep deprivation and cumulative sleep debt negatively affect performance and increase the risk of errors and accidents [8,9]. Insufficient sleep is particularly common among shift workers and has been associated with a significantly increased risk of occupational accidents compared to the recommended 7 to 8 h of sleep per night [10,11]. Several studies have reported increased rates of traffic accidents, lane deviations, and collision risks among individuals experiencing fatigue, particularly among shift workers and emergency service personnel, as well as following extended work hours or night shifts [12,13,14,15,16,17]. These findings underscore the importance of preventing drowsiness while driving as a key road safety strategy.

Given this context, detecting drowsiness in real time is therefore essential, and physiological signals offer a direct way to monitor changes in alertness. More specifically, changes in heart rate (HR), heart rate variability (HRV), and respiratory activity reflect shifts in the balance of the autonomic nervous system (ANS) that occur as alertness declines [18,19,20]. These signals are increasingly used in non-invasive and wearable monitoring systems [21].

Drowsiness is associated with measurable neurophysiological and cardiovascular changes, including alterations in HRV [22,23,24]. HRV indices, including time–domain, frequency–domain, and nonlinear measurements, have been widely used to characterize the physiological changes associated with vigilance and alertness. Rather than reflecting a single autonomic mechanism, these indices may provide complementary information about physiological changes associated with fluctuations in vigilance states [22,25]. Respiratory dynamics also change with decreasing alertness and may contribute to observed variations in HRV, although these mechanisms remain less studied [26,27,28,29]. Therefore, cardiac and respiratory signals provide a valuable physiological basis for drowsiness detection.

Existing methods for detecting drowsiness through the analysis of HR and HRV can be roughly divided into analytical and machine learning approaches. Analytical techniques rely on predefined HRV features and statistical or rule-based models to classify wakefulness and drowsiness states [25,30,31,32,33]. In comparison, machine learning methods use classifiers such as Support Vector Machine (SVM), random forests, or neural networks, trained on physiological features, to denote the complex patterns associated with drowsiness [21,26,34,35,36,37,38,39,40,41,42,43,44]. Despite these advancements, some research questions remain only partially addressed, particularly those related to ground truth labeling and subject conditioning.

In particular, the experimental design and the definition of ground truth pose major methodological challenges. In the literature, drowsiness is typically induced by sleep deprivation, the manipulation of circadian rhythms, or prolonged monotonous driving, whether in simulators or in real-world situations [21,25,30,31,32,33,34,35,38,40,41,42,43,45]. Ground truth labeling varies across studies and includes subjective measures such as the Karolinska Sleepiness Scale (KSS) or visual analog scale (VAS), psychomotor vigilance task (PVT), objective physiological markers such as sleep onset, defined via electroencephalography (EEG), and behavioral observation assessments such as the Observer Rated Drowsiness (ORD) scale or facial cues [25,30,31,33,35,37,38,42,45]. Hybrid labeling approaches further combine KSS and behavioral labels, such as PERCLOS or observer ratings, with HR-derived features, using visual indicators either as the sole ground truth or as corroborative references to validate HRV-based decision rules or classifiers [32,46]. Considering these previous studies, it is important to distinguish between studies that classify experimentally defined vigilance states and those that detect transient episodes of drowsiness using independent reference measures such as EEG, observer ratings, behavioral indicators, or subjective sleepiness scales. Although both approaches aim to characterize the decline in vigilance and alertness, they focus on different prediction targets.

Although designed to replicate real-world conditions, experimental protocols introduce variability due to laboratory and contextual factors, thereby limiting the isolation of purely physiological effects [47]. Prolonged sessions and sometimes uncontrolled factors such as caffeine intake, hydration, and meals can influence autonomic nervous system activity and perceived sleepiness, thereby confounding physiological measurements [48]. Alternatively, the constant routine, used as a reference protocol in chronobiology, isolates endogenous circadian rhythms by rigorously controlling external factors such as light, physical activity, diet, posture, and sleep–wake cycles [48,49,50]. By minimizing major behavioral and environmental confounding factors, it enhances the validity of physiological and behavioral measurements and facilitates the interpretation of circadian and sleep-deprivation-related effects on variables such as temperature, melatonin, alertness, and cognitive performance [47,50]. Data from constant-routine protocols were used to train machine learning models, such as neural networks, random forests, and XGBoost, to classify circadian phases and states of acute sleep deprivation [51,52,53]. By significantly reducing sleep–wake and behavioral masking effects, constant routines provide time series that may facilitate the classification of physiological signals while limiting several major sources of variability [54]. However, the application of machine learning classification to data from constant routine protocols remains marginal compared to general research on sleep and vigilance classification [43,55,56], and is underexplored in the context of driver drowsiness classification.

Although previous studies have applied HRV-based machine learning methods to detect driver drowsiness, most have relied on different experimental setups, including prolonged driving, real-world driving conditions, or sleep-deprivation protocols with varying levels of environmental control. Similarly, the definition of drowsiness has varied considerably from one study to another, with criteria derived from subjective assessments, behavioral observations, EEG markers, or a combination of these elements. Consequently, the direct comparison of physiological findings remains challenging, and the specific contribution of autonomic changes related to vigilance can be difficult to isolate from experimental and environmental influences. On the other hand, no study has clearly combined a controlled sleep-deprivation protocol, driving simulations, and machine-learning-based classification. The present study addresses this gap by integrating these elements into a single experimental framework.

The aim of the experimental framework is two-fold. The first aim is to improve the characterization of physiologically related changes in vigilance by reducing the influence of major behavioral and environmental confounding factors. The second aim is to provide a straightforward framework for separating experimental data into predefined Rested and Tired states under highly controlled sleep-deprivation conditions, thereby facilitating the study of physiological and behavioral changes associated with reduced vigilance. In this study, the machine learning objective is the classification of experimentally defined vigilance states. The labels “Rested” and “Tired” were derived from predefined phases of the controlled sleep-deprivation protocol and then validated using subjective, psychomotor, driving performance, and physiological measures. The study is verifying the following hypotheses:

- In a machine learning context, the integrated framework can accurately classify the experimental data into predefined Rested and Tired states, independently of measures such as KSS, VAS, and PVT.
- The separation of driving data using the integrated framework is associated with measurable differences in driving performance and allows the detection of decreased performance.
- HRV features, structured according to the integrated framework, enable machine learning models to classify states of vigilance while driving that are associated with two distinct levels of wakefulness and sleep pressure.

## 2. Materials and Methods

This study is part of a broader experimental protocol examining circadian and homeostatic variations in subjective measures of vigilance, psychomotor performance, physiological signals, and driving. Although a larger dataset was collected, including EEG, EOG, ECG, respiration, body temperature, and driving-performance measures, the present manuscript focuses specifically on an HRV-based classification of experimentally defined vigilance states during simulated driving under a highly controlled sleep-deprivation protocol.

### 2.1. Recruitment of Participants

The study protocol was conducted according to ethical and scientific approval from the Research Ethics Board of the CIUSSS de l’Ouest-de-l’Île-de-Montréal (Approval No. 2022-558, April 2022) and from the IRSST Ethics Committee (Approval No. 2020-0006, September 2022). All participants provided written informed consent prior to participation.

Healthy young adults were recruited and selected through a multi-stage screening process designed to ensure their good physical, psychological, and sleep health. Eligibility criteria included being between 18 and 30 years of age, holding a valid driver’s license for more than one year, not having worked night shifts or traveled across two or more time zones in the previous three months, and not having a history of sleep, neurological, psychiatric, or cardiovascular disorders. The screening procedures included pre-screening and screening questionnaires, clinical interviews, a medical examination, electrocardiography, blood and urine toxicology tests, and a standardized psychiatric evaluation (SCID-DSM-5) [57]. Twelve participants (five women and seven men, mean age ± SD: 24.8 ± 3.3 years) completed the entire laboratory protocol and were included in the final analyses. All female participants were using hormonal contraception and were studied during the follicular (n = 4) or luteal (n = 1) phase of their menstrual cycle.

### 2.2. Preparatory Ambulatory Period

The laboratory sessions were individually aligned to each participant’s 8-h sleep habits to preserve the alignment between the circadian system and the sleep schedule. Stabilization of the circadian system to a fixed 8 h nocturnal sleep scheduled was implemented during a week at home prior to laboratory entry. Adherence was assessed using daily sleep diaries and the continuous monitoring of physical activity and light exposure via wrist actigraphy (ActTrust, Condor Instruments Ltd.a., São Paulo, SP, Brazil).

### 2.3. Highly Controlled Laboratory Protocol

The participants were then admitted to a time-isolation laboratory at Douglas Research Centre, Montréal, Canada. On the first day, they familiarized themselves with the environment and completed two training sessions on a driving simulator. The simulator is a system developed by Professor Marie Claude Ouimet’s research team at the Faculty of Medicine and Health Sciences at the University of Sherbrooke and has been employed in several previous studies [58,59]. It consists of software (Custom driving simulation software, University of Sherbrooke, Longueuil, QC, Canada) implemented on a Windows-based computer (Windows 10/11 operating system, Microsoft Corp., Redmond, WA, USA) and displayed on three computer monitors providing frontal and peripheral views of the driving environment. Participants drove using a gas pedal, brake pedal, and steering wheel while seated in an adjustable car seat (see Figure 1).

After sensor placement, the participants underwent an eight-hour baseline sleep in total darkness (0.03 lux), monitored via polysomnography (Natus Embla NDX polysomnography system, Natus Medical Inc., Middleton, WI, USA, with SleepWorks and Harmonie software, Natus Medical Inc., Middleton, WI, USA) and visually assessed in 30-s intervals, according to the most recent American Academy of Sleep Medicine criteria [60]. At this stage, polysomnographic night recordings were used to rule out subjects with sleep disorders. After the baseline sleep period, participants slowly moved from the bed to a car seat and followed a 30-h constant routine protocol designed to minimize behavioral and environmental masking effects on the study parameters. Participants were required to remain awake in sitting position under low-light conditions (≤10 lux) at a room temperature maintained at 23.5 ± 2.0 °C and received isocaloric snacks distributed at regular hourly intervals. A brief period of standing was permitted to use the toilet every 2 h. At the end of the protocol, participants were offered an ad libitum recovery sleep opportunity. The constant-routine protocol was conducted primarily in a seated position and under highly controlled laboratory conditions. Scheduled activities, including driving simulations, psychomotor vigilance tests, and questionnaire administration, were incorporated into the protocol and standardized for all participants. Although these activities may influence physiological responses, their timing and execution were controlled to minimize additional sources of variability. Figure 2 provides an overview of the experimental framework.

### 2.4. Data Gathering

Polysomnography was recorded during sleep episodes and throughout the daily routine, including frontal, central, and occipital EEG (512 Hz), electrooculography (EOG), chin electromyography (EMG), electrocardiography (ECG) (512 Hz), and respiration (512 Hz), with standard frequency filtering. The ECG signals analyzed during the driving simulations were acquired continuously using this same polysomnography system, thereby ensuring the consistency of physiological measurements throughout all phases of the protocol. Subjective drowsiness was assessed using the KSS scale, with sleepiness operationally defined as a score ≥ 7, alongside a visual analog scale (VAS, scale from 0 to 100) measuring subjective alertness. The KSS scale and VAS were administered 3–4 times/2 h, including before and after each driving simulation. Psychomotor performance was assessed every two hours before each driving simulation using a standardized test measuring the reaction time and lapses in attention (defined as reaction times > 500 ms). Driving performance was assessed every two hours using a fixed-base, high-fidelity driving simulator equipped with standard vehicle controls and front and peripheral display screens. Each assessment consisted of a 30-min driving simulation. To minimize variability related to learning effects and task complexity, performance indicators were extracted from a standardized straight road segment at the end of each simulation. Recorded parameters included lane deviation, vehicle speed, and the steering wheel angle.

All outcome variables underwent systematic quality control prior to analysis. Subjective ratings were reviewed for data entry errors, physiological data were visually inspected for artifacts or signal loss, and driving data were checked for outliers, related to task-related deviations.

### 2.5. Data Selection

A subset of the gathered data was selected for the present project, namely those from the first and last driving simulation. At the start of the constant routine, participants were well-rested after a night of sleep and benefited from a rising circadian wake-promoting signal, resulting in low sleep pressure and high alertness. Conversely, at the end of the constant routine, participants had accumulated substantial homeostatic sleep pressure due to prolonged wakefulness (≥22 h) [61,62]. Intermediate data were not included in this analysis because they correspond to transitional periods between states of high and low vigilance. The objective of this pilot study was to evaluate the proposed framework’s ability to clearly distinguish between the “Rested” and “Tired” states. Consequently, the first and last driving simulation were selected to maximize physiological contrast while reducing uncertainty in state assignment. ECG signals were used as physiological measurements, with one 30-min ECG strip for each complete simulated drive. The subjective drowsiness ratings KSS and VAS (measured immediately after each simulated drive) and objective drowsiness metric PVT (the median reaction time measured immediately before each simulated drive) were used as variables to validate the separation between “Rested” and “Tired” states. No ECG recording was excluded from this paper because of technical issues or incomplete or unreliable data.

### 2.6. Data Processing

All calculations in this study were done with MATLAB R2024b (MathWorks Inc., Natick, MA, USA, last accessed 23 September 2026).

To enhance the R peaks and attenuate the other ECG features, each ECG signal was pre-processed with a 5th-order 5 Hz high-pass Butterworth filter, followed by a 15th-order 50 Hz finite impulse response filter with a Hamming window. All filters were applied forward and backward to implement zero-phase digital filtering.

The ECG strip for each simulated drive was segmented into 51 sections of 300 s (5 min) with a 30-s increment. The 5-min epoch allows a short-term HRV analysis. The 30-s increment fits the temporal resolution required for vigilance monitoring applications. Since the objective is to classify experimentally defined states of vigilance, HRV features were used collectively as predictor variables rather than being interpreted individually as physiological markers. For each section, R peaks were detected with a custom algorithm inspired from the Pan–Tompkins (PT) real-time ECG QRS detection algorithm [63]. RR (R peak to R peak) intervals were calculated as beat-to-beat intervals. For a specific recording, RR values deviating by more than 3 standard deviations were flagged as anomalies. RR values higher than 1500 ms (40 BPM) and lower than 500 ms (120 BPM) were also considered anomalies. Ectopic beats were not specifically addressed in this study. The flagged RR interval outliers were simply removed. R-peak detections were then visually inspected. No imputation or replacement of abnormal RR intervals was performed. Based on our decision to include only RR intervals of 40 to 120 BPM, we assumed that each window of 300 s should have between 200 and 600 RR intervals. All 300-s windows containing less than 200 or more than 600 RR intervals were considered to respectively have gaps or false detections and excluded from HRV calculations. HRV parameters were calculated based on the remaining normal intervals/windows after the reported abnormalities were removed. Furthermore, no participants or ECG recordings were excluded due to missing or incomplete data. This preprocessing approach ensured that only valid normal-to-normal (NN) intervals were used to calculate the downstream 27 HRV features (see Table 1). In the present study, the RR interval limits of 500 ms and 1500 ms were used as conservative preprocessing criteria for this cohort of healthy young adults, studied under a highly controlled constant-routine protocol. The aim was not to define absolute physiological boundaries, but rather to minimize the influence of potential detection errors and artifactual intervals on the HRV estimation. These criteria were applied uniformly across all recordings and vigilance states studied. Although wider limits have been used in studies involving different populations or more demanding cardiovascular conditions, the selected thresholds here were deemed appropriate for the standardized laboratory setting and the objectives of a pilot study in the present investigation.

### 2.7. Data Analysis

Statistical comparisons between the Rested and Tired states were performed using a subject-wise approach. For each variable, two paired observations were available per participant, corresponding to the first and last simulated drives. These observations were grouped into Rested and Tired states, yielding a nominal sample size of 12 paired observations. Differences between conditions were assessed using a two-sided paired Wilcoxon signed-rank test, which tested the null hypothesis that paired differences originated from a distribution with a zero median. Statistical significance was set at *p* < 0.05. The effect size was quantified using Cohen’s dz:

(1) $$dz=\;\frac{Mean(d)}{SD(d)}$$

where *d* denotes the difference between paired observations. Effect sizes were interpreted as large (|*dz*|≥ 0.80), medium (0.50 ≤ |*dz*| < 0.80), or small (0.20 ≤ |*dz*| < 0.50).

For KSS, VAS, and PVT, a single value was recorded for each participant during each simulated drive and used directly in the analysis.

Driving-performance variables were obtained as 4 min time series for each simulated drive. The signals were segmented into 48 non-overlapping 5-s windows, and the standard deviation within each window was computed to quantify driving variability. Window-level values were then averaged to obtain a single value per participant and drive. Prior to averaging, outliers associated with potential roadway departures or other highly erratic driving episodes were removed, as these events may reflect driving skill rather than the vigilance state.

For HRV analysis, 51 values were obtained per participant per simulated drive for each feature. Feature values were averaged across each drive to generate a single value for each vigilance state. Raw HRV features are reported in the descriptive statistics. However, statistical comparisons (*p* values and dz) were conducted using the preprocessed HRV features employed for machine-learning model development (see Section 2.8).

### 2.8. Data Classification

A subject-wise approach was also used for machine learning. The Leave-One-Subject-Out Cross-Validation (LOSO-CV) model testing method was selected to train 4 classification models on HRV features: AdaBoost (AB), k-nearest neighbors (KNN), random forest (RF), and Support Vector Machine (SVM). When applying the LOSO-CV to the dataset of 12 participants, the data was divided into 12 independent iterations. In each iteration, the data from 11 participants were used to form the training set, while the data from the remaining participant were reserved exclusively for testing.

To enhance class separability, raw HRV features were transformed using a combined symmetrical logarithmic and inverse hyperbolic sine scaling procedure, defined as asinh (symlog (feature value)). Subject-level z-score normalization was subsequently applied. Each feature comprised 1224 epochs, corresponding to 12 subjects × 2 simulated drives per subjects × 51 epochs per drive. The 612 epochs from the first simulated drive were labeled as Rested, whereas the 612 epochs from the last simulated drive were labeled as Tired.

All data-dependent model development procedures were performed exclusively using the training data from each LOSO iteration. Specifically, for each iteration, the held-out subject was first excluded from the dataset. Feature ranking and selection using the Minimum Redundancy Maximum Relevance(MRMR) algorithm, as well as hyperparameter optimization, were then performed using the data from the remaining 11 training subjects only. The resulting subset of features and the optimized model configuration were then used for the held-out participant. Since scaling/normalization was performed independently for each participant, no information was shared between the training and the testing participants during preprocessing. This procedure ensured complete separation between training and testing participants and prevented information leaks throughout the model development and evaluation. The process was repeated until each participant had served as a test participant exactly once, and the resulting performance metrics were averaged across the 12 LOSO iterations.

The performance of trained models was evaluated with the calculation of accuracy, sensitivity (recall), specificity, precision, and F1 score, defined as follows:

(2) $$Accuracy=\;\frac{TP+TN}{TP+TN+FP+FN}$$

(3) $$Sensitivity\;(Recall)=\frac{TP}{TP+FN}$$

(4) $$Specificity=\frac{TN}{TN+FP}$$

(5) $$Precision=\frac{TP}{TP+FP}$$

(6) $$F1\;score=2\times \frac{Precision\times Recall}{Precision+Recall}$$

True positives (*TP*s) are all the epochs correctly identified as Tired. False positives (*FP*s) are the epochs incorrectly identified as Tired. The *TN* and *FN* are respectively the true negative and false negative.

## 3. Results

### 3.1. State Separation Validated by Subjective Ratings and Psychomotor Performance

To validate state separation by drowsiness ratings and reaction times, KSS, VAS, and PVT paired values were dispatched into Rested and Tired states for each subject and compared (see Figure 3 and Table 2). The Tired state had higher KSS ratings, lower VAS ratings, and higher reaction times. All differences were significant, with large effect sizes. It is also interesting to notice higher standard deviations and ranges for the Tired state.

### 3.2. State Separation Validated by Driving Behavior

To validate state separation by driving behavior, the paired standard deviations of lane position, vehicle speed, and steering wheel position were dispatched into Rested and Tired states for each subject and compared (see Figure 4 and Table 3). The Tired state had higher variabilities for all three variables. All differences were significant, with a large effect size. It is also interesting to notice consistently higher standard deviations and ranges for the Tired state.

### 3.3. State Separation Validated by HRV

To validate state separation by HRV, the paired values of the 27 HRV features were dispatched into Rested and Tired states for each subject and compared (see Table 4, Table 5 and Table 6). It is important to note that numbers in those tables only provide a descriptive characterization of differences across the 27 extracted HRV variables rather than testing predefined clinical assumptions regarding individual HRV features. According to Table 6, across all features, HRV variables vary 15% on average from Rested to Tire states. The changes are significant for 41% of the features, with medium to large effect sizes for 48% of the features. The averaged coefficient of variation (CV) being above 0.4 indicates that the data is highly scattered around the mean, even if the spread diminishes from the Rested to Tired state. Time–domain features display better performance compared to their frequency and nonlinear counterparts.

### 3.4. State Detection with Machine Learning

The results of machine learning demonstrated that HRV features extracted from the experimentally defined Rested and Tired states enabled a reliable and independent classification of participants under the Leave-One-Subject-Out Cross-Validation (LOSO-CV) framework. As shown in Figure 5 and Table 7, all four classifiers achieved high classification performance, with average accuracies ranging from 86.36% (KNN) to 91.26% (AdaBoost). AdaBoost achieved the highest overall accuracy, sensitivity, specificity, precision, and F1 score, with respective values of 91.26%, 89.22%, 90.66%, 93.55%, and 90.77%. Random forest also showed high performance, with an average accuracy of 90.28% and a precision of 94.50%. For all classifiers, sensitivity and specificity remained well balanced, indicating comparable reliability in identifying both Rested and Tired states. Consistent with these results, ROC curves (Figure 6) and confusion matrices (Figure 7) demonstrated robust discrimination capabilities for all evaluated models.

Subject-level accuracies (Table 8) confirmed that the classification framework generalized well across participants, with average accuracy rates exceeding 85% for most participants. Although classification performance varied from one individual to another, particularly for subjects 8 and 11, the overall results of the LOSO cross-validation remained stable and confirmed the models’ ability to generalize to previously not-retained participants.

The feature-subset analysis performed using the random forest classifier (Table 9) revealed that time–domain HRV features achieved the highest classification performance among all the feature groups evaluated. The subset of the time domain yielded an average accuracy of 90.28%, outperforming both the complete set of 27 features (85.21%) and the Top09 subset selected by MRMR (86.27%), the features from the nonlinear domain (83.66%), and the features from the frequency domain (75.08%). These results indicate that time–domain HRV features contained the most discriminative information for distinguishing between Rested and Tired states in the present dataset. Consequently, the time–domain HRV feature set was retained as the primary feature subset for participant-independent classification of the vigilance state in the present study.

Additional machine learning results obtained using the Top09 subset selected by the MRMR algorithm are presented in Appendix A (Figure A1, Figure A2 and Figure A3 and Table A1). Although the subset selected by MRMR improved performance compared to the complete set of 27 features, the time–domain HRV features achieved the highest participant-independent classification performance, supporting their selection as the primary feature subset in this study.

## 4. Discussion

In this study, we presented a single experimental framework integrating a highly controlled 30-h sleep-deprivation protocol, simulated drives, and machine-learning-based classification, to study changes in vigilance and driving performances during a prolonged period of wakefulness.

The data were first dispatched into two experimentally defined conditions, Rested and Tired states, corresponding to the first and the last driving sessions of the protocol. The first and last driving sessions represent the two observations furthest apart in time within the protocol and thus offer the sharpest contrast in terms of cumulative wakefulness for each participant. This approach preserves the within-subject nature comparison of the study while avoiding the additional dependency structure introduced by combining multiple repeated sessions within the same condition. Then, state separation was validated with subjective ratings (KSS and VAS), objective psychomotor performance (PVT), and driving performance. The differences between Rested and Tired states were significant for the KSS, VAS, and PVT, with large effect sizes, confirming that the protocol successfully induced two distinct states of vigilance. Driving performance also deteriorated significantly under the Tired state, as evidenced by increased variability in the lane position, vehicle speed, and steering wheel angle. These results indicate that the experimentally defined states were associated with measurable changes in subjective alertness, psychomotor performance, and driving behavior.

As the objective of the present study was a physiological classification rather than investigation of the underlying autonomic mechanisms, individual HRV features were interpreted as markers associated with vigilance states rather than as direct indicators of specific sympathetic or parasympathetic activity. The Rested and Tired states analyzed in this study correspond to predefined phases of a prolonged wakefulness protocol. Therefore, the observed differences between these states may reflect the combined influence of accumulated sleep pressure, the circadian rhythm, repeated controlled exposure to a task, and other time-dependent factors. This framework was designed to distinguish between experimentally defined states of vigilance rather than isolating the specific contribution of a particular physiological mechanism. Accordingly, the present findings demonstrate distinctions between experimentally defined vigilance states under highly controlled laboratory conditions and should not be interpreted as direct evidence of drowsiness detection while driving in real-world situations, which would require independent external validation.

According to our analysis, several HRV features showed significant differences between Rested and Tired states, with effect sizes ranging from moderate to large observed for some time–domain, frequency–domain, and nonlinear features. These results suggest that physiological changes related to vigilance can be captured through HRV analysis under highly controlled experimental conditions. At the same time, the observed alterations were not uniformly distributed across all HRV features, and considerable interindividual variability persisted. This heterogeneous pattern may help explain some of the inconsistencies reported in previous HRV-based drowsiness and vigilance studies, especially as these discrepancies could be mainly due to methodological differences and uncontrolled confounding factors. Some studies were conducted under conditions where external factors such as caffeine consumption, dietary intake, hydration, posture, and light exposure were not strictly controlled, even though they are known to influence autonomic nervous system activity and HRV measures [47,48]. Furthermore, previous methods used to induce drowsiness vary considerably from one study to another, with some relying on sleep deprivation, others on prolonged monotonous driving, and still others on natural driving conditions, each involving different physiological processes and levels of fatigue and drowsiness [25,30,31,33,34,42,43,45]. The drowsiness definition also varies considerably, as some studies use subjective measures such as the KSS scale, others rely on EEG-based physiological markers, and still others use behavioral or contextual indicators, leading to inconsistencies in the interpretation of changes in HRV [30,31,35,37,38,42]. On the other hand, interindividual variability and differences in validation strategies can contribute further to the variability in the results [38,42]. Overall, these factors limit the ability to isolate the specific contribution of physiological changes related to vigilance to HRV in previous studies.

Machine learning analyses demonstrated that the physiological changes observed between the Rested and Tired states could be effectively captured using HRV metrics. Although the extent of the changes varied across HRV features, the highest participant-independent classification performance was obtained using the time–domain HRV feature subset. This result suggests that time–domain measures contained the most discriminative information for distinguishing between the two experimentally defined vigilance states in the present study. The classification performance achieved in this study was comparable to that reported in previous studies using similar HRV-based machine learning approaches [37,42,43] and supports the feasibility of HRV-based participant-independent classification of vigilance states under controlled sleep-deprivation conditions.

To our knowledge, our study is the first to have clearly combined a highly controlled sleep-deprivation protocol, simulated drives, and machine-learning-based classification into a single experimental framework. By controlling for the masking effects of light exposure, postural changes, and food intake, this study helps characterize the HRV differences observed between experimentally defined Rested and Tired states, as well as their correlation with driving performances.

There are differences in the timing of variables relative to one another: KSS and VAS were recorded immediately after each drive, the PVT median reaction time immediately before each drive, and driving-performance metrics from a standardized straight-road segment at the end of each drive. However, those differences are unlikely to affect study interpretation because comparisons were conducted within each variable only (Rested vs. Tired), rather than across variables, to verify state separation during the early and late phases of the 30-h wakefulness protocol. Similarly, HRV features were derived from the full 30-min drive and compared only between Rested and Tired states to track vigilance.

The relatively small sample size of the participant cohort is reflecting the logistical and ethical constraints inherent to human circadian studies. Although participant-independent validation was performed using a LOSO-CV approach and the classification models demonstrated robust performance for most participants, the limited number of independent subjects remains a significant constraint on the generalizability of the findings. Statistical analyses were also conducted using the participant as the independent experimental unit, with paired comparisons made between the first and last driving sessions to provide a more rigorous assessment of differences between the Rested and Tired states. Nevertheless, future studies involving larger cohorts could strengthen the statistical characterization of changes related to vigilance and allow for the use of more advanced modeling approaches, such as mixed-effects models that explicitly account for repeated measurement patterns.

The present study focused on classifying the experimentally defined Rested and Tired states and therefore did not examine the continuous evolution of vigilance throughout the 30-h protocol. Although the selection of the first and last simulated driving sessions of the protocol was intended to maximize the physiological contrast between states, future studies could incorporate all repeated assessments to characterize the trajectory of changes in vigilance and determine whether significant transition points occur during prolonged wakefulness.

In this work, only HRV characteristics were used for classification. Other physiological signals collected during the protocol, such as respiration and EEG, were deliberately excluded to restrict the scope of the study but could provide complementary or synergistic information. Although respiration was recorded during the protocol, respiratory variables were not included in the present analyses. The potential influence of respiration on HF-related HRV metrics, respiratory sinus arrhythmia (RSA)-related measures, and other frequency–domain HRV indices cannot be excluded. In future studies, the integration of both respiratory and cardiorespiratory coupling metrics could improve sensitivity to intersubject fluctuations in vigilance and enhance the reliability of classification. Future research could further explore the methodological comparison by evaluating other classification approaches, including Logistic Regression as a baseline model and more advanced gradient boosting methods such as XGBoost. Such comparisons could help determine whether the observed classification performance is primarily driven by the physiological information contained in the HRV features or to the specific learning algorithm used. Alternatively, the use of deep learning architectures, such as convolutional or recurrent neural networks, could exploit the temporal dependencies of HRV dynamics more effectively than classical classifiers.

Future studies could also examine the use of highly immersive virtual reality environments based on head-mounted displays to assess whether greater immersion influences physiological responses, driving behavior, and the classification of vigilance levels. Finally, driving performance was evaluated in a simulated environment, which does not allow for the complete reproduction of the requirements of driving in real conditions. To transpose this framework to real-time embedded physiological monitoring systems in the vehicle, it will be necessary to solve the problems related to signal quality, computational efficiency, and individualized calibration, for which the vigilance detection models are adapted to the basic physiological characteristics and autonomic response profile of each driver.

## 5. Conclusions

In this pilot study, we provided preliminary evidence that combining a standardized protocol, driving simulation, and machine learning provides a useful framework for investigating changes in vigilance during prolonged wakefulness situations. By minimizing major behavioral and environmental confounding factors, we found that the experimentally defined Rested and Tired states during a prolonged wakefulness protocol were associated with significant changes in multiple HRV features, as a marked decline in subjective alertness, psychomotor performance, and driving performance.

Although not all HRV features contributed equally to the separation of states, reliable participant-independent classification of the experimentally defined Rested and Tired states was obtained under the LOSO-CV validation, with the highest performance achieved using time–domain HRV features. This result highlights the feasibility of using controlled experimental protocols and multivariate analysis of HRV for studying vigilance-related physiological alterations.

Overall, this work supports the development of non-invasive, physiology-based alertness monitoring systems for road safety applications. Future studies involving larger and more diverse populations will be required to evaluate the generalizability of the presented findings.

## Figures and Tables

**Figure 1:**
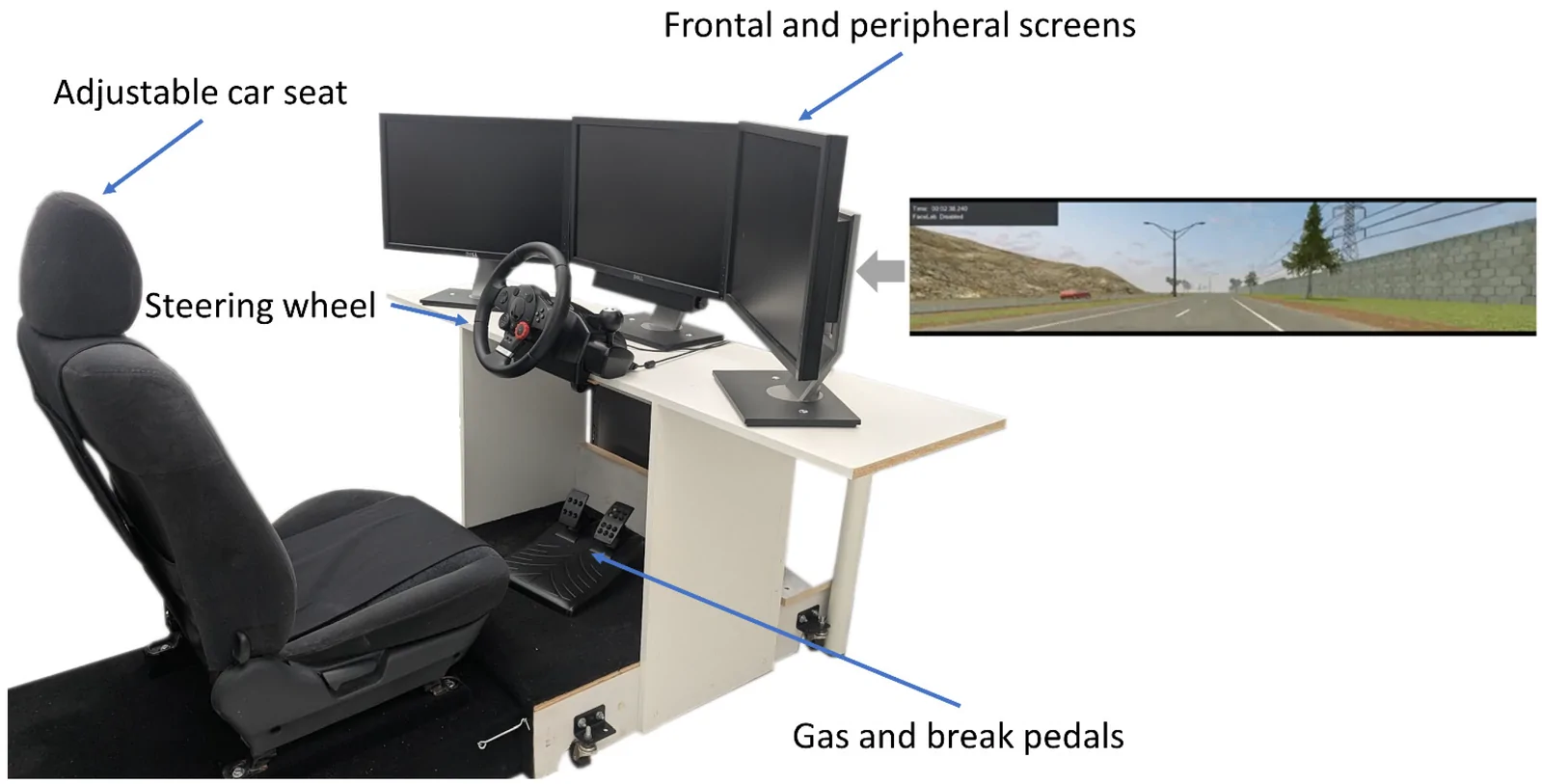
Driving simulation setup.

**Figure 2:**
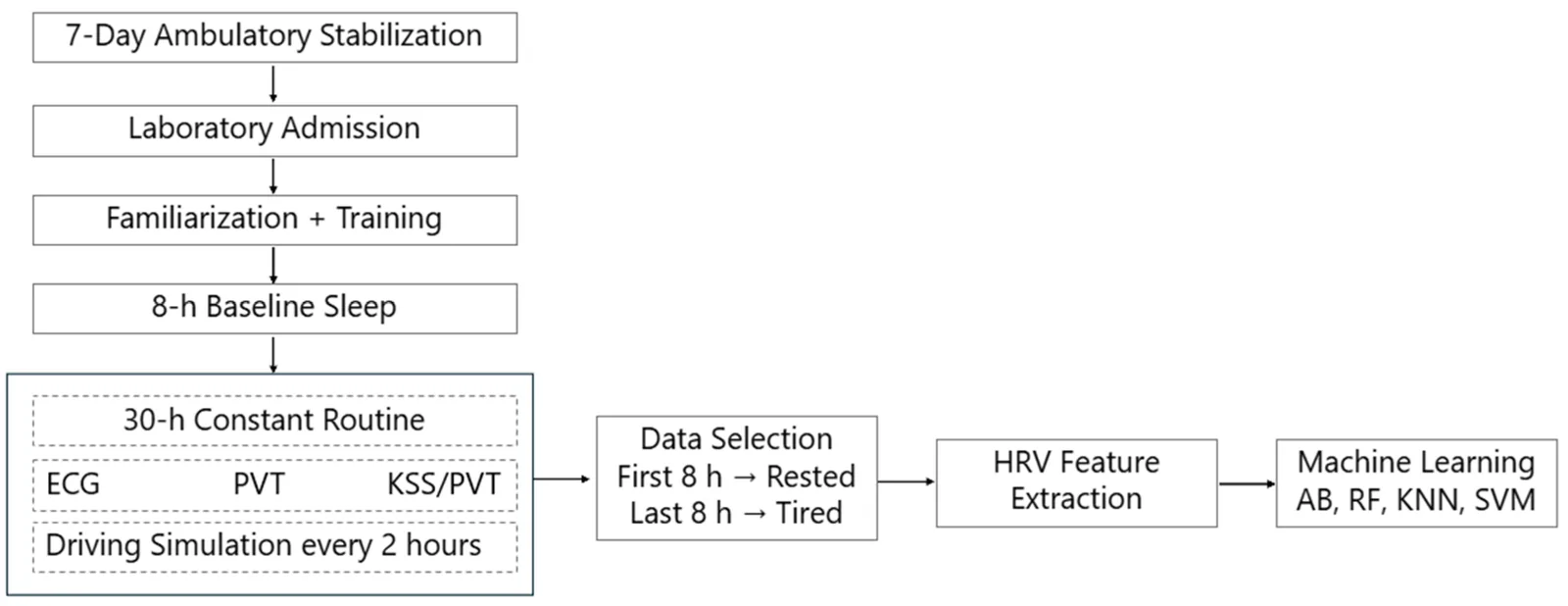
Graphical overview of the experimental framework.

**Figure 3:**
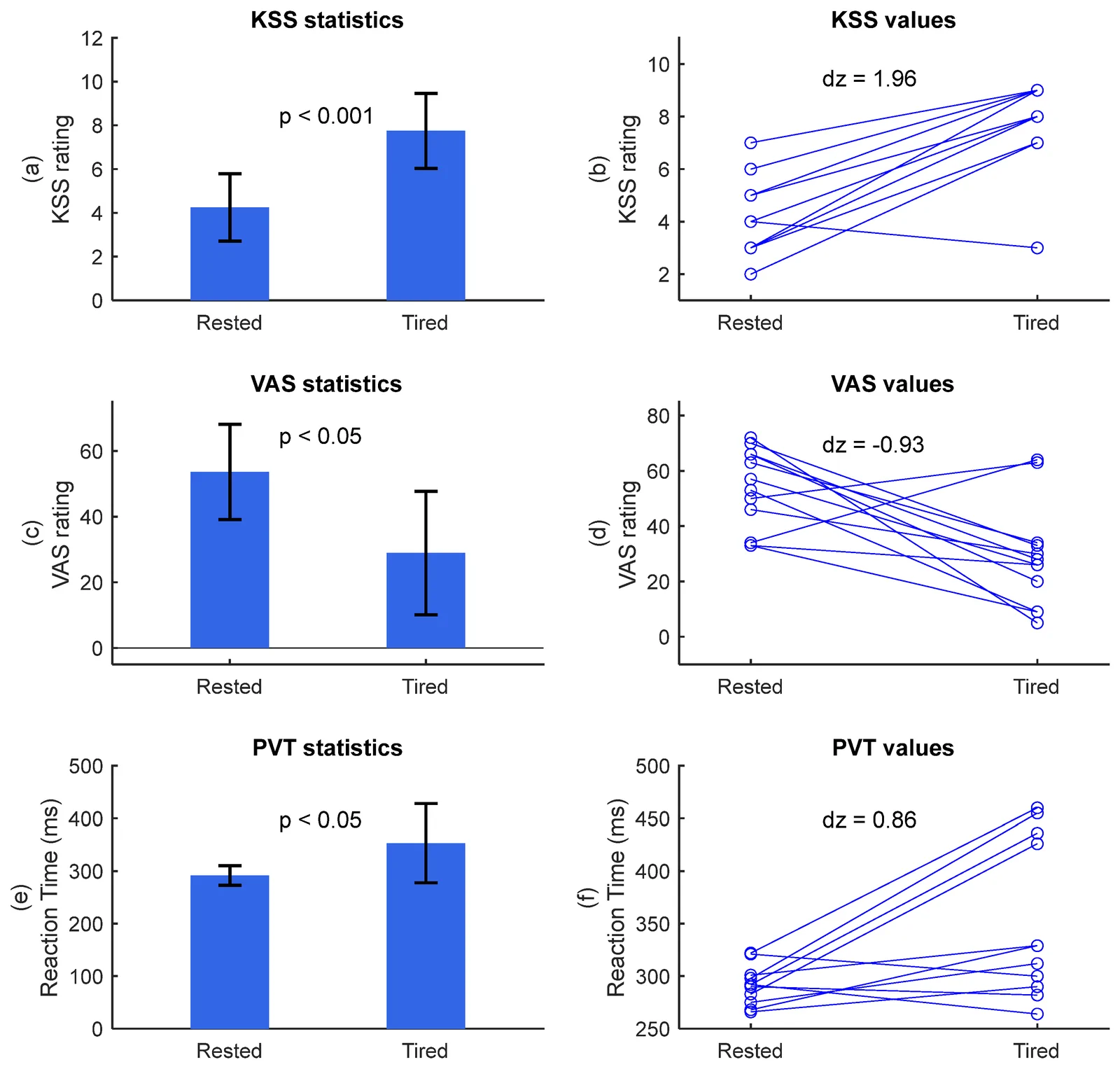
State separation validated by subjective ratings and the psychomotor vigilance test. (**a**,**c**,**e**) Bar plots showing the averages of KSS, VAS, and PVT values: Rested vs. Tired. The average is taken over 12 subjects for KSS and VAS and 11 subjects for PVT. The error bars are standard deviations. (**b**,**d**,**f**) Paired data plot for KSS, VAS, and PVT: Rested vs. Tired. Each line represents one subject. Each dot is the value collected from one subject in a Rested or Tired state. The average value of the dots in a state is used to display the corresponding bar plot.

**Figure 4:**
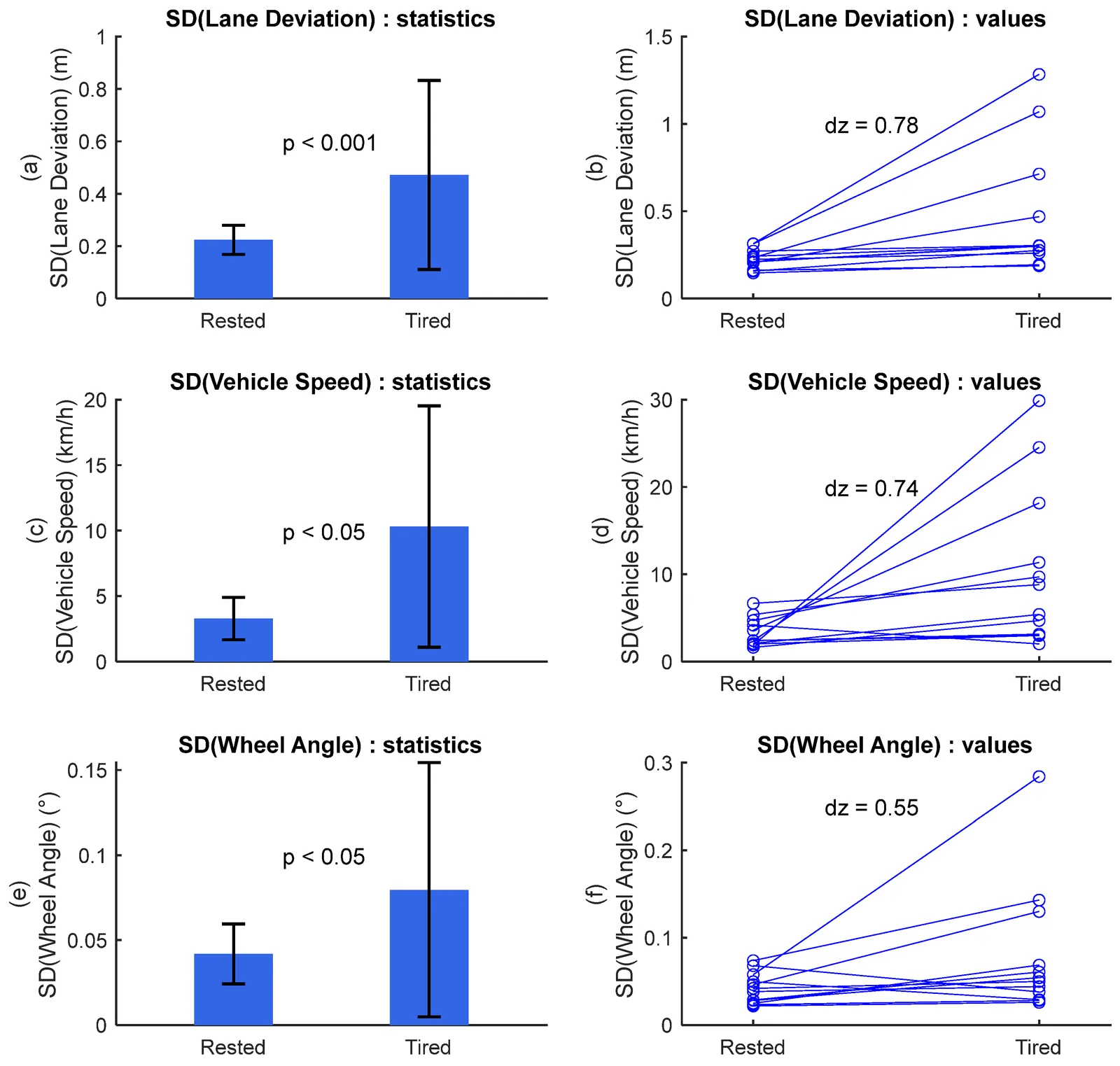
State separation validated by driving behavior performance. (**a**,**c**,**e**) Bar plots showing average variability of lane deviations, vehicle speeds, and steering wheel angles: Rested vs. Tired. The average is taken over 12 subjects. The error bars are standard deviations. (**b**,**d**,**f**) Paired data plot for the variability of lane deviations, vehicle speeds, and steering wheel angles: Rested vs. Tired. Each line represents one subject. Each dot is the value collected from one subject in the Rested or Tired state. The average value of the dots in a state is used to display the corresponding bar plot.

**Figure 5:**
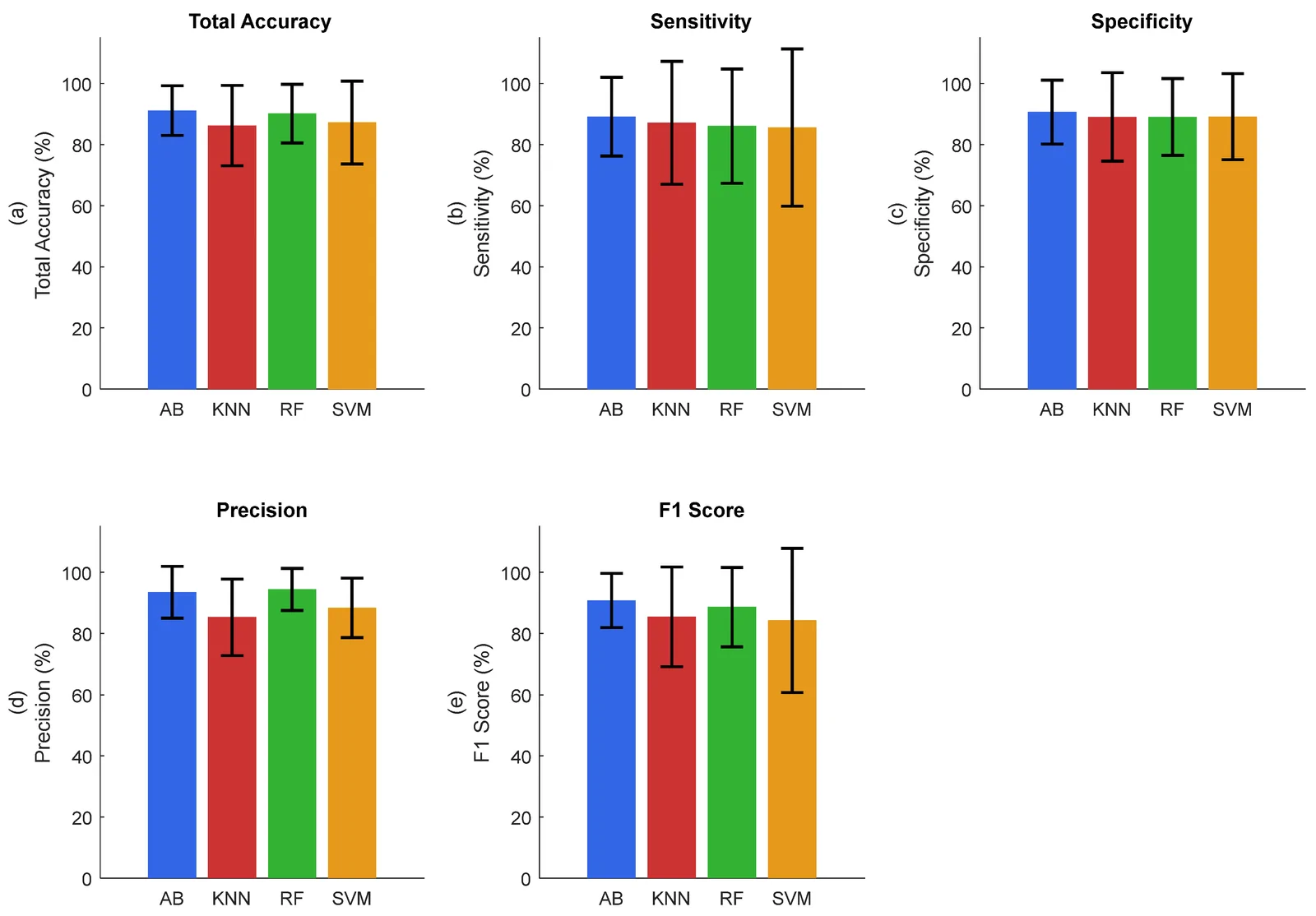
Binary classification performance for four algorithms.

**Figure 6:**
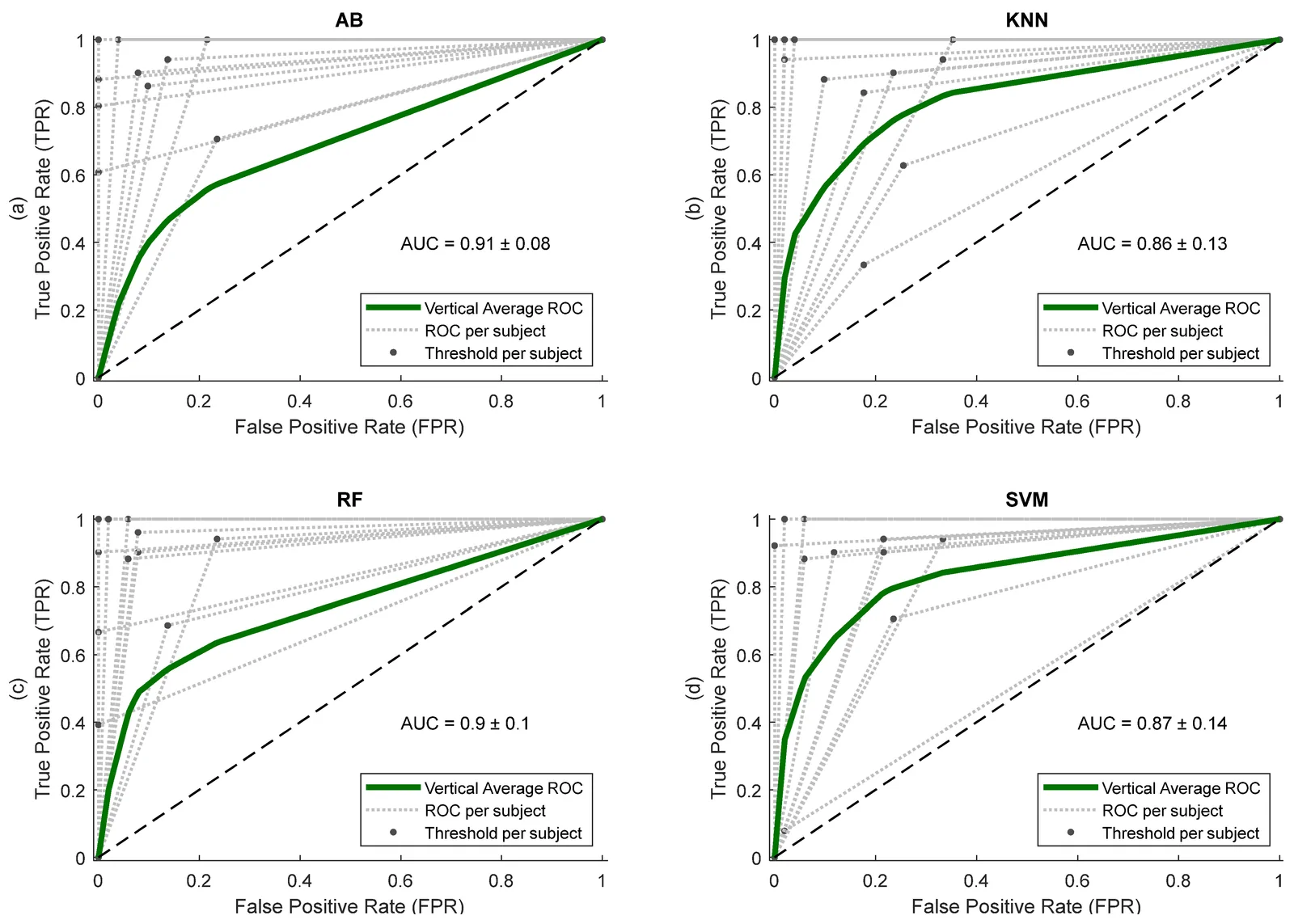
ROC curves characterizing the binary classification performance of the four models.

**Figure 7:**
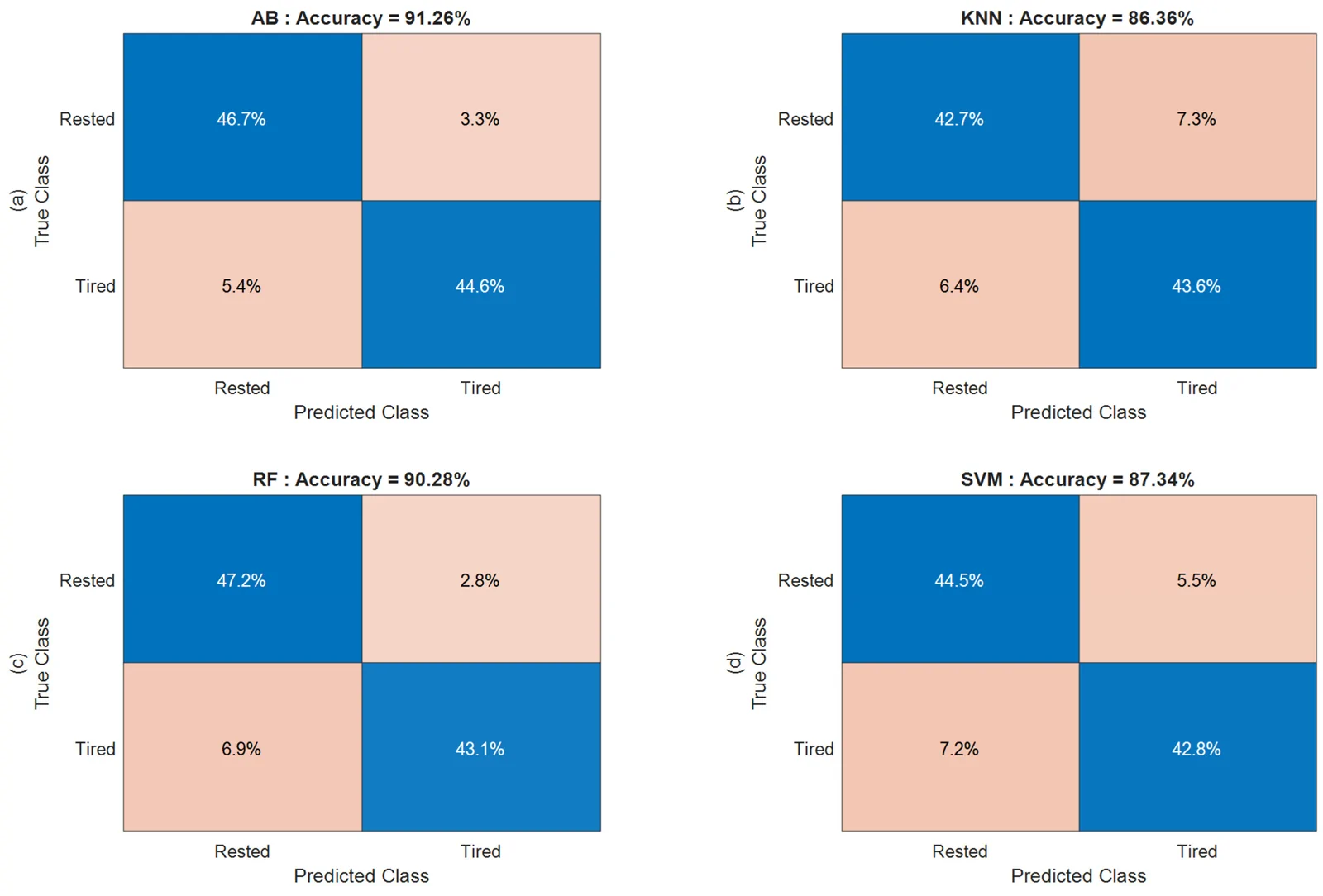
Confusion matrices characterizing the binary classification performance of the four models.

**Table 1:** HRV features (27): categories, abbreviations, and descriptions.

Category	Abbreviation	Description
Time domain	RangeNN	Range of NN intervals
	MeanNN	Mean of NN intervals
	SDNN	Standard deviation of NN intervals
	CVNN	Coefficient of variation of NN intervals
	RMSSD	Root mean square of successive differences
	pNN50	Percentage of NN intervals differing by more than 50 ms
	HTI	HRV Triangular Index of the NN interval histogram
	TINN	Triangular Interpolation of the NN interval histogram
	BSI	Baevsky’s stress index of the NN interval histogram
Frequency domain	TP	Total frequency power (0 Hz–0.4 Hz)
	LF	Low-frequency power: Power (0.04 Hz–0.15 Hz)
	HF	High-frequency power: Power (0.15 Hz–0.4 Hz)
	LFnu	Normalized LF: 100% × LF/(LF + HF)
	HFnu	Normalized HF: 100% × HF/(LF + HF)
	LF/HF	Ratio of LF to HF: LF/HF
	LFpeak	Peak frequency of LF
	HFpeak	Peak frequency of HF
Nonlinear domain	SampEn	Sample entropy
	SD1	Standard deviation of the points perpendicular to the line of identity (y = x) of a Poincaré plot
	SD2	Standard deviation of the points along the line of identity (y = x) of a Poincaré plot
	SDA	Area of the ellipse = pi × SD1 × SD2
	SD1/SD2	Ratio of SD1 to SD2: SD1/SD2
	DFA α1	Short-term scaling exponent from detrended fluctuation analysis
	RCR	Recurrence rate: Density of recurrence points from recurrence quantification analysis
	DET	Determinism: Proportion of recurrence points that form diagonal lines from recurrence quantification analysis
	ShanEn	Shannon entropy: The Shannon entropy of the probability distribution of the diagonal line lengths from recurrence quantification analysis
	LAM	Laminarity: Proportion of recurrence points that form vertical lines from recurrence quantification analysis

**Table 2:** State separation validated by subjective ratings and psychomotor performance. n is the number of subjects. For PVT, there was no data collected for subject #2.

	KSS		VAS		PVT Reaction Time (ms)	
	Rested	Tired	Rested	Tired	Rested	Tired
Median	4	8	55	27	292	329
Mean	4.25	7.75	54	29	292	353
SD	1.55	1.71	15	19	19	75
Range	5	6	39	59	56	196
n	12	12	12	12	11	11
*p*	9.77 × 10^−4^		1.22 × 10^−2^		2.05 × 10^−2^	
dz	1.96		−0.93		0.86	

**Table 3:** Sleep-deprivation assessment with driving behavior. n is the number of subjects.

	SD of Lane Position (m)		SD of Vehicle Speed (km/h)		SD of Steering Wheel Position (°)	
	Rested	Tired	Rested	Tired	Rested	Tired
Median	0.217	0.302	2.4	7.1	0.040	0.052
Mean	0.224	0.472	3.27	10.3	0.042	0.080
SD	0.055	0.361	1.62	9.21	0.018	0.075
Range	0.167	1.10	5.04	28	0.052	0.258
n	12	12	12	12	12	12
*p*	4.88 × 10^−4^		4.88 × 10^−3^		3.42 × 10^−2^	
dz	0.78		0.74		0.55	

**Table 4:** Sleep-deprivation assessment with HRV: statistics. The mean and SD are calculated over the 12 subjects. The values of *p* and dz are calculated, not with the raw HRV data, but with the HRV data that was preprocessed and normalized for machine learning.

	Mean Rested	Mean Tired	*p*	dz	SD Rested	SD Tired
**RangeNN (ms)**	364	436	0.18	0.54	210	190
**MeanNN (ms)**	776	800	0.18	0.31	106	91
**SDNN (ms)**	69	83	0.01	0.83	43	34
**CVNN**	0.086	0.103	0.02	0.90	0.047	0.038
**RMSSD (ms)**	53	67	0.02	0.87	50	41
**pNN50 (%)**	19	30	0.02	0.89	19	18
**HTI**	15	17	0.02	0.88	5.96	4.52
**TINN (ms)**	230	268	0.02	0.73	94	68
**BSI (s^−2^)**	103	55	0.15	−0.59	92	42
**TP (ms^2^)**	6043	7511	0.02	0.80	7646	6071
**LF (ms^2^)**	1854	2345	0.06	0.64	2207	1785
**HF (ms^2^)**	1823	2010	0.02	0.76	3143	2213
**LFnu (%)**	63	59	0.20	−0.33	13.43	11.89
**HFnu (%)**	37	41	0.20	0.33	13.43	11.89
**LF/HF**	2.11	1.65	0.30	−0.30	1.25	0.79
**LFpeak (Hz)**	0.053	0.046	0.38	−0.36	0.018	0.011
**HFpeak (Hz)**	0.263	0.240	0.37	−0.26	0.059	0.065
**SampEn**	1.30	1.32	0.73	0.15	0.146	0.140
**SD1 (ms)**	38	47	0.02	0.86	35	29
**SD2 (ms)**	90	108	0.02	0.85	52	41
**SDA (ms^2^)**	15,501	19,273	0.01	0.90	22,783	19,522
**SD1/SD2**	0.375	0.410	0.30	0.36	0.123	0.113
**DFA α1**	1.62	1.59	0.52	−0.20	0.110	0.117
**RCR**	0.057	0.055	0.73	−0.16	0.008	0.006
**DET**	0.788	0.781	0.42	−0.25	0.022	0.023
**ShanEn**	1.67	1.64	0.79	−0.15	0.128	0.116
**LAM**	0.752	0.757	0.91	0.02	0.072	0.061

**Table 5:** Sleep-deprivation assessment with HRV: analysis of the statistics. |Δ| is the relative variation of the mean HRV value from the Rested to Tired state, with the Tired state as the reference value. Statistically significant HRV variations (*p* < 0.05 from Table 4) are set to 1. HRV relative variations with a medium effect size or higher (dz ≥ 0.5 from Table 4) are set to 1. SD values are replaced with the coefficient of variation (CV = SD/Mean) to allow cross-state and cross-feature comparisons.

	|Δ| (%)	*p* < 0.05	dz ≥ 0.5	CV Rested	CV Tired
**RangeNN (ms)**	16.35	0	1	0.58	0.44
**MeanNN (ms)**	2.96	0	0	0.14	0.11
**SDNN (ms)**	17.15	1	1	0.62	0.41
**CVNN**	16.32	1	1	0.54	0.36
**RMSSD (ms)**	20.20	1	1	0.93	0.61
**pNN50 (%)**	35.52	1	1	0.99	0.60
**HTI**	14.99	1	1	0.40	0.26
**TINN (ms)**	14.06	1	1	0.41	0.25
**BSI (s^−2^)**	88.34	0	0	0.89	0.77
**TP (ms^2^)**	19.54	1	1	1.27	0.81
**LF (ms^2^)**	20.94	0	1	1.19	0.76
**HF (ms^2^)**	9.29	1	1	1.72	1.10
**LFnu (%)**	6.59	0	0	0.21	0.20
**HFnu (%)**	9.36	0	0	0.36	0.29
**LF/HF**	27.53	0	0	0.59	0.48
**LFpeak (Hz)**	14.26	0	0	0.35	0.23
**HFpeak (Hz)**	9.94	0	0	0.22	0.27
**SampEn**	1.72	0	0	0.11	0.11
**SD1 (ms)**	20.14	1	1	0.93	0.61
**SD2 (ms)**	16.72	1	1	0.58	0.38
**SDA (ms^2^)**	19.57	1	1	1.47	1.01
**SD1/SD2**	8.44	0	0	0.33	0.27
**DFA α1**	1.77	0	0	0.07	0.07
**RCR**	2.84	0	0	0.15	0.11
**DET**	0.78	0	0	0.03	0.03
**ShanEn**	1.68	0	0	0.08	0.07
**LAM**	0.69	0	0	0.10	0.08

**Table 6:** Sleep-deprivation assessment with HRV: interpretation of the statistics. The percentage pct of features with *p* < 0.05 and dz ≥ 0.5 is calculated. The other columns are averages from Table 5.

	Mean (|Δ|) (%)	pct (*p* < 0.05) (%)	pct (dz ≥ 0.5) (%)	Mean (CV) Rested	Mean (CV) Tired
**All**	15.47	40.74	48.15	0.57	0.40
**Time Domain**	25.10	66.67	77.78	0.61	0.42
**Frequency Domain**	14.68	25.0	37.50	0.74	0.52
**Nonlinear Domain**	7.44	30.0	30.0	0.38	0.27

**Table 7:** Descriptive statistics of the LOSO-CV classification performance for the AB, KNN, RF, and SVM models.

Model	Indices	Mean	SD	Min	Max
**AB**	Accuracy	91.26	8.15	73.53	100
	Sensitivity	89.22	12.97	60.78	100
	Specificity	90.66	10.47	71.83	100
	Precision	93.55	8.44	75.00	100
	F1 Score	90.77	8.96	72.73	100
**KNN**	Accuracy	86.36	13.21	57.84	100
	Sensitivity	87.25	20.07	33.33	100
	Specificity	89.10	14.47	55.26	100
	Precision	85.40	12.44	65.38	100
	F1 Score	85.56	16.26	44.16	100
**RF**	Accuracy	90.28	9.61	69.61	100
	Sensitivity	86.11	18.72	39.22	100
	Specificity	89.14	12.48	62.20	100
	Precision	94.50	6.81	80.00	100
	F1 Score	88.78	12.86	56.34	100
**SVM**	Accuracy	87.34	13.59	52.94	99
	Sensitivity	85.62	25.83	7.84	100
	Specificity	89.10	14.17	51.55	100
	Precision	88.48	9.78	73.85	100
	F1 Score	84.38	23.49	14.29	99

**Table 8:** Binary classification accuracy per subject. At the end of each line is the average accuracy of a specific subject across all models.

Subject	AB	KNN	RF	SVM	Average
1	100	100	100	99.02	99.75
2	89.22	82.35	91.18	80.39	85.78
3	90.20	83.33	94.12	89.22	89.22
4	90.20	96.08	83.33	96.08	91.42
5	94.12	89.22	95.10	91.18	92.40
6	100.00	99.02	100	97.06	99.02
7	98.04	98.04	97.06	99.02	98.04
8	80.39	57.84	69.61	52.94	65.20
9	100	98.04	99	99.02	99.02
10	88.24	80.39	91.18	84.31	86.03
11	73.53	68.63	77.45	73.53	73.28
12	91.18	83.33	85.29	86.27	86.52

**Table 9:** Random forest model accuracy for different feature selections: all features, Top09 of this paper selected with the MRMR algorithm and time–domain (TD), frequency–domain (FD), and nonlinear domain (NLD) features. At the end of each line is the average RF accuracy of a specific subject across all categories of feature selection. At the bottom of each column is the average RF accuracy of a specific feature selection category across all subjects.

Subject	All	Top09	TD	FD	NLD	Average
**1**	100	100	100	100	99.02	99.80
**2**	58.82	74.51	91.18	58.82	55.88	67.84
**3**	93.14	89.22	94.12	84.31	86.27	89.41
**4**	84.31	85.29	83.33	77.45	90.20	84.12
**5**	93.14	92.16	95.10	93.14	95.10	93.73
**6**	100	99	100	96.08	100	99.02
**7**	96.08	89.22	97.06	86.27	93.14	92.35
**8**	65.69	70.59	69.61	40.20	75.49	64.31
**9**	100	98	99.02	98.04	99.02	98.82
**10**	78.43	90.20	91.18	50.00	59.80	73.92
**11**	66.67	66.67	77.45	55.88	61.76	65.69
**12**	86.27	80.39	85.29	60.78	88.24	80.20
**Average**	85.21	86.27	90.28	75.08	83.66	

**Figure A1:**
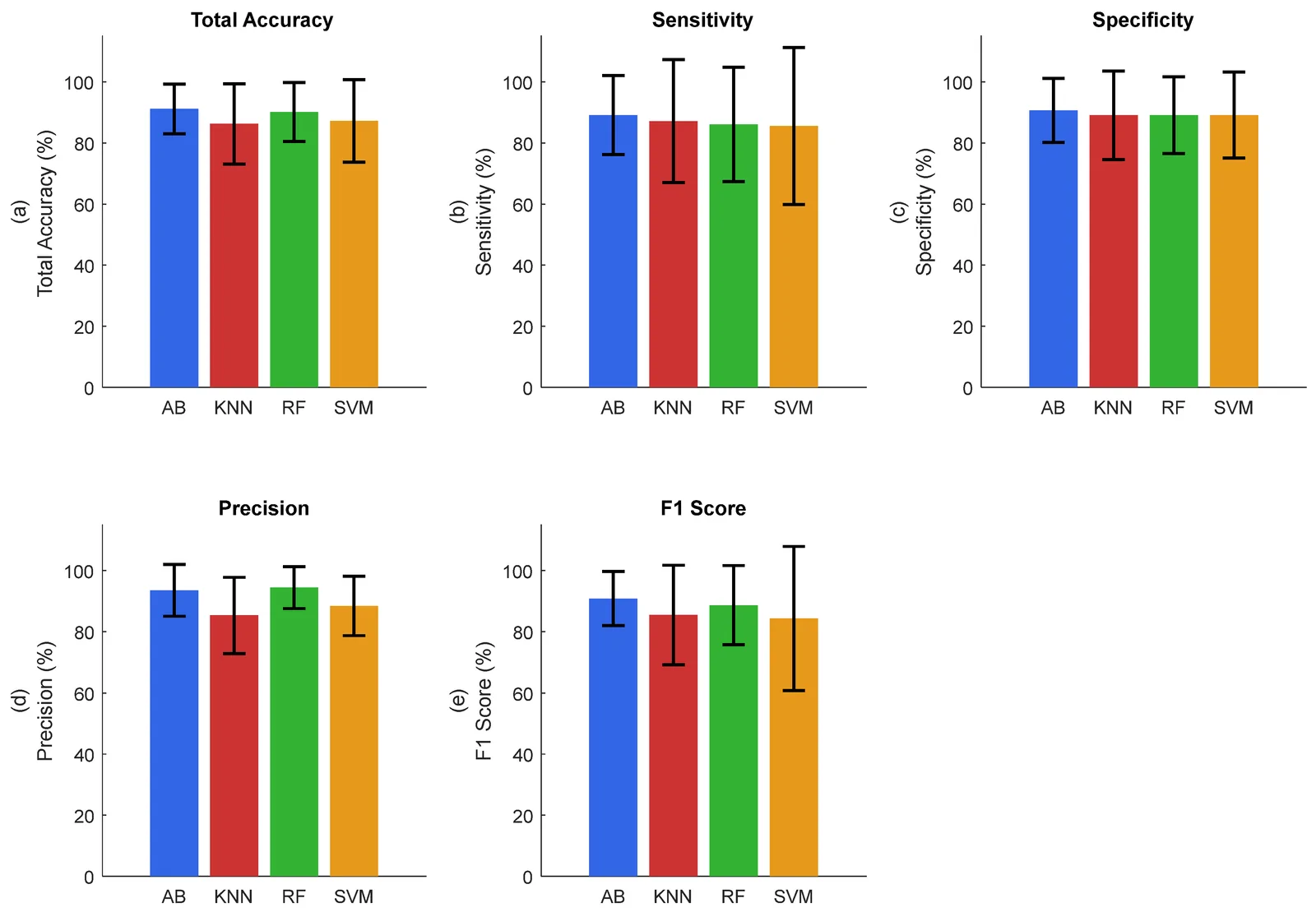
Binary classification performance of four algorithms: Top09 features selected with MRMR algorithm.

**Figure A2:**
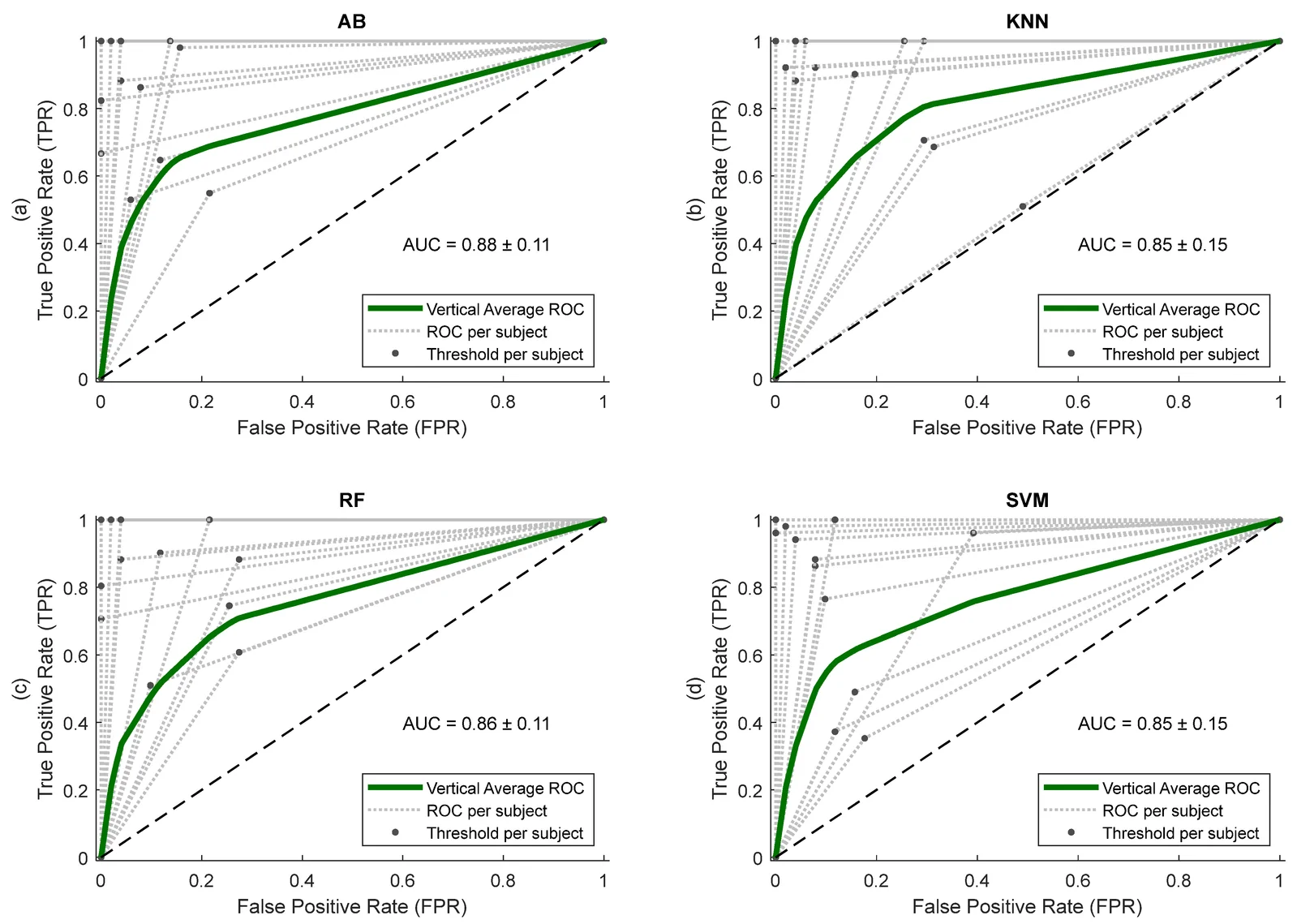
ROC curves characterizing the binary classification performance of the four models: Top09 features selected with MRMR algorithm.

**Figure A3:**
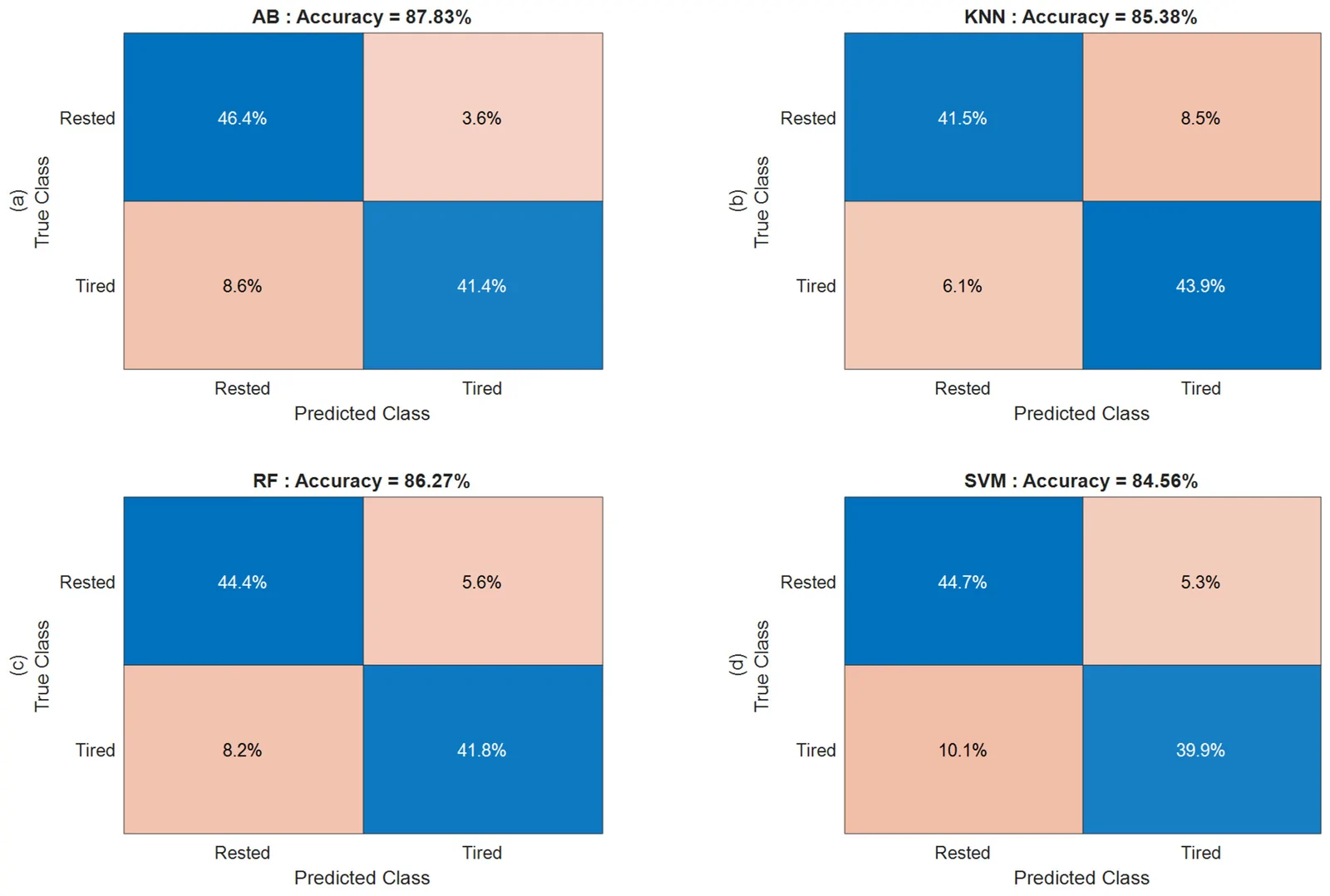
Confusion matrices characterizing the binary classification performance of the four models: Top09 features selected with MRMR algorithm.

## Data Availability

The raw physiological data were collected at the Centre for Study and Treatment of Circadian Rhythms, Douglas Mental Health University Institute, Department of Psychiatry, McGill University. For ethical, confidentiality, and participant-consent restrictions, the authors cannot provide public access to these data. The MATLAB scripts, trained models, and associated analysis resources developed for this project are currently not available for public distribution. Nevertheless, the manuscript provides a detailed description of the signal processing pipeline, HRV feature extraction procedures, statistical analyses, and the machine learning methodology to facilitate the independent implementation and scientific evaluation of the proposed framework. However, the authors agree to make data and materials supporting the results or analyses available for the investigation of scientific integrity if necessary.

## Appendix A. Binary Classification Performance for Top09 Features Selected with the MRMR Algorithm

**Table A1:** Descriptive statistics of the LOSO-CV classification performance for the AB, KNN, RF, and SVM models using the Top09 HRV feature subset selected by the MRMR algorithm.

Model	Indices	Mean	SD	Min	Max
**AB**	Accuracy	87.83	10.66	66.67	100
	Sensitivity	82.84	18.38	52.94	100
	Specificity	86.29	13.97	63.49	100
	Precision	91.86	8.41	71.79	100
	F1 Score	86.31	12.78	62.22	100
**KNN**	Accuracy	85.38	14.73	50.98	100
	Sensitivity	87.75	15.98	50.98	100
	Specificity	87.80	15.98	50.98	100
	Precision	84.07	15.03	50.98	100
	F1 Score	85.65	14.75	50.98	100
**RF**	Accuracy	86.27	11.12	66.67	100
	Sensitivity	83.66	16.54	50.98	100
	Specificity	85.85	13.20	64.79	100
	Precision	88.69	11.24	68.89	100
	F1 Score	85.40	12.46	63.41	100
**SVM**	Accuracy	84.56	14.63	58.82	100
	Sensitivity	79.74	24.76	35.29	100
	Specificity	84.52	16.57	56.00	100
	Precision	87.09	11.72	66.67	100
	F1 Score	81.99	19.28	46.15	100
